# Risk Factors for Post-Stroke Pneumonia in a Patient Population with Subacute Stroke: A Retrospective Cohort Study

**DOI:** 10.3390/jcm12185835

**Published:** 2023-09-08

**Authors:** Hiroyuki Tashima, Mari Ito, Michiyuki Kawakami, Ryota Ishii, Yuta Miyazaki, Tomonori Akimoto, Masahiro Tsujikawa, Keigo Kobayashi, Kunitsugu Kondo, Tetsuya Tsuji

**Affiliations:** 1 Department of Rehabilitation Medicine, Tokyo Bay Rehabilitation Hospital, Chiba 275-0026, Japan; 2 Department of Rehabilitation Medicine, Keio University School of Medicine, Tokyo 160-8582, Japan; 3 Department of Biostatistics, Faculty of Medicine, University of Tsukuba, Ibaraki 305-8577, Japan; 4 Department of Radiology, Yatsu Hoken Hospital, Chiba 275-0026, Japan

**Keywords:** subacute stroke, post-stroke pneumonia, rehabilitation, predictors, comprehensive rehabilitation ward

## Abstract

The risk of pneumonia and death is higher in acute stroke patients with signs of pulmonary infection on chest computed tomography (CT) at admission. However, few reports have examined the incidence of pneumonia and its predictors in subacute stroke patients. The aim of this study was to examine factors related to post-stroke pneumonia in subacute stroke patients. A total of 340 subacute stroke patients were included. Univariable logistic regression analysis was performed using variables that may contribute to pneumonia, with the development of pneumonia as the dependent variable. Multivariable logistic regression analysis using the three independent variables with the lowest *p*-values on the univariable logistic regression analysis was also performed to calculate adjusted odds ratios. Twenty-two patients developed pneumonia during hospitalization. The univariable logistic regression analysis showed that the top three items were serum albumin (Alb), functional Oral Intake Scale (FOIS) score, and signs of pulmonary infection on chest CT at admission. Multivariable logistic regression analysis adjusted for these three items showed that the presence of signs of pulmonary infection on chest CT at admission was the independent variable (OR: 4.45; 95% CI: 1.54–12.9). When signs of pulmonary infection are seen on admission chest CT, careful follow-up is necessary because pneumonia is significantly more likely to occur during hospitalization.

## 1. Introduction

Post-stroke pneumonia occurs in approximately 12.3% of acute stroke patients [1], and it is associated with increased mortality [2], higher medical costs [3], and decreased activities of daily living (ADL) [4]. Inflammation, including infection, also contributes to stroke risk through a variety of interrelated mechanisms [5]. Chronic respiratory, urinary, dental, and other infections amplify the risk of developing carotid atherosclerosis [6]. Elevated levels of the inflammatory mediators interleukin (IL)-6, C-reactive protein (CRP), and lipoprotein-associated phospholipase A (Lp-PLA2) are associated with increased stroke risk [7]. In addition, chronic active inflammation has been reported to have a significant impact on the progression and impact of stroke risk factors. Therefore, research on prevention and the factors related to post-stroke pneumonia is important.

A systematic review and meta-analysis examining predictors of the development of post-stroke pneumonia in acute stroke patients found that age, male sex, smoking, severity according to the National Institutes of Health Stroke Scale (NIHSS), presence of dysphagia, use of a nasogastric tube, ventilator management, diabetes mellitus, chronic obstructive pulmonary disease, and atrial fibrillation were identified as predictors [8]. In addition to this, it has been reported that the risk of pneumonia and death was higher when chest computed tomography (CT) at admission within 24 h of stroke onset showed signs of pulmonary infection [9], suggesting that the imaging findings may be factors related to post-stroke pneumonia. Because of this possibility, this report concluded that early administration of antibiotics to a patient with signs of pulmonary infection on admission chest CT may lead to better outcomes [9]. On the other hand, to the best of our knowledge, no reports have examined the incidence of post-stroke pneumonia and predictors of pneumonia in subacute stroke patients admitted to a comprehensive rehabilitation ward.

The Stroke Recovery and Rehabilitation Roundtable developed the framework defining what is meant by “acute,” “sub-acute,” and “chronic,” building on previous work. In the roundtable, time from stroke onset 7 days–3 months was classified as “Early subacute,” and 3–6 months was classified as “Late subacute” [10]. In Japan, there are comprehensive rehabilitation wards called Kaifukuki Rehabilitation Wards (KRWs) [11]. A KRW is a system for rehabilitation during the subacute phase and is covered by government medical insurance. Patients are admitted to an acute care hospital after stroke onset and generally receive one to two months of treatment. After the acute phase treatment, the patients are admitted to a comprehensive rehabilitation ward when the medical staff judges that their condition is stable and ready for rehabilitation. Stroke patients with severe disabilities and cognitive disorders could stay for up to 180 days. The prevalence of dysphagia in stroke patients on rehabilitation wards has been reported to range from 28% to 59% [12], with a certain number of patients developing post-stroke pneumonia. In addition, it has been reported that post-stroke pneumonia is associated with improved ADL [13]. Thus, it is important to investigate the factors related to post-stroke pneumonia in patients with subacute stroke.

The aim of this study was to retrospectively examine the factors, including chest CT, related to post-stroke pneumonia in subacute stroke patients admitted to a comprehensive rehabilitation ward.

## 2. Materials and Methods

### 2.1. Study Design

This was an observational, retrospective study carried out in accordance with the Declaration of Helsinki with the approval of the Tokyo Bay Rehabilitation Hospital’s Institutional Ethics Review Board (286-2). This study was conducted from 31 March 2022, when the Ethics Review Board gave its approval. Informed consent was obtained in the form of opt-out on the Tokyo Bay Rehabilitation Hospital’s website.

### 2.2. Participants

This study included subacute stroke patients admitted to the Tokyo Bay Rehabilitation Hospital after being treated at an acute care hospital from 13 May 2020 to 30 November 2021. The inclusion criteria were as follows: (1) first admission (i.e., not including readmissions), and (2) chest CT was performed on admission. The exclusion criteria were as follows: (1) not having been diagnosed by a radiologist, and (2) more than 6 months passed since the onset at the time of hospitalization (i.e., subacute stroke patients were selected). All patients were followed-up until they were transferred or discharged. Two patients were excluded because the attending physician did not ask the radiologist to diagnose the CT images. This was not due to poor image quality or adverse outcomes. One patient was excluded because six months had passed since the onset of stroke at the time of admission. Therefore, the remaining 340 patients were evaluated.

### 2.3. Study Setting

This study was conducted in the Tokyo Bay Rehabilitation Hospital, which serves as a KWRs, with 160 beds. Patients underwent daily one-on-one intensive rehabilitation sessions with therapists for approximately 120–180 min a day during the hospitalization. The typical contents of rehabilitation were ≥60 min of physical therapy, ≥60 min of occupational therapy, and/or ≥60 min of speech–language–hearing therapy. Rehabilitation consisted mainly of repetitive, task-specific training, aimed to improve paralysis and reacquiring activities of daily living. Speech–language–hearing therapy training included feeding and swallowing training. If dysphagia was suspected, swallowing function could be examined by videofluorography (VF).

### 2.4. Data Acquisition

Data on factors that could affect pneumonia during hospitalization, including age, sex, stroke type, duration from stroke onset to admission to our hospital, day of onset of pneumonia, length of stay in our hospital (the observation period for the development of pneumonia), body temperature, blood test data such as white blood count (WBC), CRP, and serum albumin (Alb), height, weight, body mass index (BMI) at admission, functional Oral Intake Scale (FOIS) score [14], Stroke Impairment Assessment Set (SIAS) [15], Functional Independence Measure motor (m-FIM)/Functional Independence Measure cognitive (c-FIM) scores [16] at admission and discharge, and FIM effectiveness [17], were extracted from medical records retrospectively.

The FOIS score has a 7-grade scale of oral intake to evaluate swallowing function. Scores less than 3 points indicate an enteral-tube-dependent individual, whereas scores of more than 4 points indicate an individual who has achieved total oral intake. The FOIS score may be appropriate for evaluating and documenting changes in the functional eating abilities of stroke patients over time [14].

The FIM is a comprehensive measure of ADL from both physical and cognitive perspectives. It is widely accepted as being used to measure functional abilities in patients undergoing rehabilitation. The FIM consists of 13 motor subscales (m-FIM) and 5 cognitive subscales (c-FIM). The m-FIM consists of the following four categories: self-care (eating, grooming, bathing, dressing-upper body, dressing-lower body, and toileting), sphincter control (bladder management and bowel management), transfers (bed, chair, wheelchair, toilet, tub, and shower), and locomotion (walk, wheelchair, and stairs). The c-FIM consists of two categories: communication (comprehension and expression) and social cognition (social interaction, problem solving, and memory). Each item has a 7-grade scale ranging from 1 (total assistance or not testable) to 7 points (complete independence). The total score is 13–91 points, and 5–35 points for the total m-FIM and c-FIM. A higher score represents greater functional independence.

The SIAS is a comprehensive assessment index consisting of 22 motor and sensory items that has been validated for reliability and validity in post-stroke disability. The SIAS Motor consists of two tests of the upper extremity (knee-mouth and finger function tests) and three tests of the lower extremity (hip flexion, knee extension, and foot pat tests). Each test was rated on a 6-grade ordinal scale rating from 0 (no movement at all) to 5 points (normal). The total scores of the upper and lower extremities were 0–10 and 0–15 points.

In this study, the SIAS used to assess motor paralysis and the FIM to assess functional independence in ADL were used to reflect the severity level of the stroke patients. All CT images were diagnosed by the same radiologist with more than 15 years of clinical experience who is qualified as a radiation specialist in Japan. The presence of signs of pulmonary infection on chest CT at admission was defined as the presence of consolidation, ground-glass opacity, or nodular shadow (imaging findings suggestive of pneumonia rather than clinically evident pneumonia). Post-stroke pneumonia during hospitalization was defined as cases meeting the following criteria that were treated with antibiotics. Both of the following criteria were met: (i) alveolar infiltration shadow on chest X-ray or chest CT; and (ii) the presence of any two or more of the following: temperature above 37.5 °C, high serum CRP, WBC count ≥ 9000/μL, and airway symptoms such as sputum [18]. Only the presence of pneumonia was confirmed, and the type of pneumonia, such as bacterial or viral, was not specified. Authors could access information that could identify individual participants during or after data collection.

### 2.5. Statistical Analysis

Quantitative variables are summarized by mean (standard deviation; SD) and median (interquartile range; IQR, 25–75th percentiles) values, whereas categorical variables are summarized by frequencies and proportions (%). Univariable logistic regression analyses were performed with pneumonia as the dependent variable. In addition, multivariable logistic regression analysis was performed with the three items showing the lowest *p*-values on the univariable regression as independent variables. All statistical analyses were performed using IBM SPSS Statistics 27.

## 3. Results

Patients’ baseline characteristics are shown in Table 1. This study included patients with ischemic stroke, intracerebral hemorrhage, and subarachnoid hemorrhage. The results of chest CT on admission are shown in Table 2. Of the 340 patients, 222 (65.3%) had abnormal shadows, 30 (8.82%) had signs of pneumonia, 129 (37.9%) had parenchymal changes after pneumonia, 29 (8.53%) had pleural effusions, 75 (22.1%) had atelectasis, and 20 (5.88%) had emphysema. No patients with signs of pneumonia on chest CT at admission met the definition of developing pneumonia when the chest CT was performed. Although there were signs of pneumonia on imaging, they were asymptomatic and did not require antibiotic treatment. Patient characteristics based on comparison of the two groups of patients with and without signs of pneumonia at admission are listed in the Supplementary Materials.

On the other hand, 22 patients (6.47%) developed pneumonia during hospitalization. In the univariable logistic regression analysis with pneumonia onset during hospitalization as the dependent variable, the top three items with the lowest *p*-values were FOIS score, Alb, and signs of pneumonia on chest CT at admission (Table 3); therefore, these items were used in the multivariable logistic regression analysis. The FOIS score, Alb, and signs of pneumonia on chest CT at admission were significant independent variables in the binomial logistic regression analysis (Table 4) (OR: 4.45; 95% CI: 1.54–12.9).

## 4. Discussion

On our comprehensive rehabilitation ward in a hospital for subacute stroke patients, pneumonia occurred in 6.47% (22/340) of the patients during their hospitalization. The FOIS score, Alb, and signs of pneumonia on chest CT at admission were factors related to the development of pneumonia.

Prior studies have reported that two of three cases of post-stroke pneumonia within 3 months of stroke onset occurred in the first week, with peak onset on the third day [19]. The Pneumonia in Stroke Consensus (PISCES) group has proposed the term stroke-associated pneumonia (SAP) to encompass all terms referring to lower respiratory tract infections in stroke patients within 7 days after stroke onset [20]. It has been reported that stroke-induced immunosuppression (SIIS), or a series of processes in which the immune system is suppressed in the periphery after stroke onset, is an important factor in SAP development [21]. In the present study, a certain number of patients developed pneumonia even after the high-risk phase for post-stroke pneumonia, when the aforementioned mechanisms that occur in the acute phase are not likely to be involved. Therefore, it is important to investigate the factors related to post-stroke pneumonia in subacute stroke patients admitted to a comprehensive rehabilitation ward.

The analysis identified the FOIS score, Alb, and signs of pneumonia on chest CT at admission as factors related to the development of pneumonia in patients with subacute stroke.

In one report, stroke patients with dysphagia had a ≥3-fold increase in risk of pneumonia. They also reported an 11-fold increase in risk in a subset of more severely impaired patients with confirmed aspiration [22]. Therefore, the association of dysphagia assessed by the FOIS score in the present study with the development of pneumonia is consistent with these previous studies. The presence of a nasogastric tube (NGT) may also affect bacterial colony formation through biofilm formation on the tube [23], making it more likely to cause gastroesophageal reflux and vomiting [24]. Aspiration of bacterial-laden secretions or infected reflux increases the risk of pneumonia [25]. In the FOIS score used in the present study, users of gastroduodenal tubes were classified as Level I-III, which accounted for 16.5% (56/340) of the study’s population. It is possible that, in addition to dysphagia, the gastric tube may have contributed to the development of pneumonia.

The serum albumin level has been used for the diagnosis of protein–energy malnutrition [26] and malnutrition (evaluated by mini nutritional assessment and BMI); the serum albumin level is strongly associated with oropharyngeal dysphagia in older patients hospitalized for an acute disease [27]. There is also a report that the serum albumin level is an independent predictor of nosocomial pneumonia [28], and that the admission serum albumin level shows a significant negative correlation with the number of infective complications in acute stroke patients [29]. The present study suggests that serum albumin levels are associated with the occurrence of pneumonia even in subacute stroke patients.

In our comprehensive rehabilitation ward, more than half of the patients with subacute stroke at admission had abnormal shadows in the lung fields. This was clearly more common than in a Canadian study of healthy individuals (68.0 ± 9.0 years) with no smoking history (11.8% had findings of bronchiolitis) [30]. This may be due to the fact that stroke patients are more susceptible to pneumonia in the acute phase and are more likely to have a smoking history and underlying medical conditions than healthy subjects. In addition, 8.82% of patients (30/340) had imaging findings suggestive of pneumonia, but no patients with findings suggestive of pneumonia met the diagnostic criteria for pneumonia at admission. Considering the high incidence of pneumonia in the acute phase and the fact that the patients were asymptomatic despite findings suggestive of pulmonary infection, it is possible that these CT results reflect the healing process after acute pneumonia. Aspiration can also cause lung inflammation, but in most cases, the clinical course is self-limited and may have been asymptomatic.

Of the patients who had imaging findings suggestive of pneumonia, 26.7% (8/30) developed clinically evident pneumonia during the hospitalization period, and the time it took them to develop pneumonia was 24.4 ± 21.4 days. Since there was variation among patients, and some cases developed pneumonia several weeks after admission, careful follow-up is necessary if there are findings suggestive of pneumonia on chest CT.

The mechanism by which pneumonia develops in the subacute phase may differ from that of pneumonia occurring in the acute phase (SAP) [20,21], but, to the best of our knowledge, few studies have reported the predictors and incidence of pneumonia in this phase. The strong point of this study is that, in addition to common predictors of pneumonia, imaging findings at admission were used to study the disease. We believe that this may be a new factor related to the development of pneumonia and is very significant for future research.

The limitations of this study include that it was a single-center study, and thus the results must be generalized with caution. In addition, detailed information on factors such as whether the patient had developed pneumonia or had a fever requiring antibiotic therapy during previous hospitalization as a factor related to the presence of pneumonia at the time of admission was scarce and could not be inferred. Regarding the type of pneumonia that occurred during the hospitalization, it was not possible to determine whether it was bacterial or viral because sputum culture and viral tests were not performed. As for the statistical analysis, the number of independent variables in the logistic regression analysis could not be increased due to the small number of patients who developed pneumonia or were transferred to other hospitals during the hospitalization period; therefore, further accumulation of cases is necessary. Despite these limitations, we believe that the present findings provide important information for the study of predictors of the development of pneumonia in patients with subacute stroke.

## 5. Conclusions

The present results suggest that the serum albumin level and the FOIS score, which was evaluated as a swallowing-related factor, were associated with the development of pneumonia not only in acute stroke patients, but also in subacute stroke patients. Chest CT at admission may be a new factor related to the development of pneumonia. If we identify signs of pulmonary infection on admission chest CT, careful follow-up is necessary, because some cases developed pneumonia several weeks after admission.

## Figures and Tables

**Table 1:** Characteristics of the participants and variables as factors predicting post-stroke pneumonia.

	Overall (n = 340)
Age (y)	70.6 (14.2)
Sex (male) (%)	201 (59.1)
Type of stroke (%)	
Ischemic	215 (63.2)
Hemorrhagic	110 (32.4)
Subarachnoid hemorrhage	15 (4.4)
Duration from stroke onset to admission (days)	34.1 (21.3)
Length of stay (days)	83.9 (51.9)
Functional Independence Measure motor at admission	41.0 (21.3)
Functional Independence Measure cognitive at admission	20.5 (8.76)
Stroke Impairment Assessment Set at admission	15.5 (8.87)
Functional Oral Intake Scale score at admission	5 (5–7)
Body mass index (kg/m^2^)	21.8 (3.52)
Body temperature (°C)	36.5 (0.41)
White blood count (/μL)	6845.5 (2250.6)
C-reactive protein (mg/dL)	0.79 (1.93)
Serum albumin (mg/dL)	3.82 (0.46)

Quantitative variables are summarized as mean (standard deviation; SD) and median (interquartile range; IQR, 25–75th percentiles) values. Categorical variables are summarized as frequencies and percentages.

**Table 2:** Abnormal shadows on chest CT at admission.

	Overall (n = 340)
Abnormal shadows, n (%)	222 (65.3)
Signs of pneumonia, n (%)	30 (8.82)
Consolidation	10
Ground-glass opacity	19
Nodular shadow	7
Parenchymal changes after pneumonia, n (%)	129 (37.9)
Pleural effusion, n (%)	29 (8.53)
Atelectasis, n (%)	75 (22.1)
Emphysema, n (%)	20 (5.88)

**Table 3:** Univariable logistic regression analysis with pneumonia onset during hospitalization as the dependent variable.

	Crude Odds Ratio	95% CI	*p*-Value
Mean age	1.05	1.01–1.09	1.59 × 10^−2^
Sex (male)	4.73	1.37–16.3	1.38 × 10^−2^
Duration from stroke onset to admission	1.02	1.00–1.03	3.76 × 10^−2^
Length of stay	1.00	0.99–1.01	7.02 × 10^−1^
Functional Independence Measure motor at admission	0.92	0.88–0.96	1.05 × 10^−4^
Functional Independence Measure cognitive at admission	0.88	0.83–0.94	1.06 × 10^−4^
Stroke Impairment Assessment Set at admission	0.94	0.89–0.98	5.60 × 10^−3^
Functional Oral Intake Scale score at admission	0.61	0.49–0.75	3.00 × 10^−6^
Body Mass Index at admission	0.86	0.74–1.00	4.49 × 10^−2^
White blood count at admission	1.00	1.00–1.00	4.36 × 10^−1^
C-reactive protein at admission	1.24	1.09–1.42	1.58 × 10^−3^
Serum albumin at admission	0.12	0.05–0.31	1.60 × 10^−5^
Signs of pneumonia at admission	7.69	2.91–20.3	3.80 × 10^−5^

**Table 4:** Multivariable logistic regression analysis for pneumonia onset during hospitalization.

	Crude Odds Ratio	95% CI	*p*-Value
Functional Oral Intake Scale score at admission	0.70	0.89–0.98	6.10 × 10^−3^
Serum albumin at admission	0.21	0.07–0.68	8.89 × 10^−3^
Signs of pneumonia at admission	4.45	1.54–12.9	5.84 × 10^−3^

## Data Availability

The present research involved human research participant data, which raises ethical issues regarding the protection of personal information, and we have not received approval to publicly share our data from the Ethics Committee. However, the datasets used and analyzed during the current study are available from the corresponding author on reasonable request with permission from the Ethics Committee of the Tokyo Bay Rehabilitation Hospital. Researchers can contact the Ethics Committee of the Tokyo Bay Rehabilitation Hospital by email or letter. The email of the Ethics Committee of the Tokyo Bay Rehabilitation Hospital is shinsakai@wanreha.net. The mailing address of the Ethics Committee of the Tokyo Bay Rehabilitation Hospital is 4-1-1 Yatsu, Narashino, Chiba, Japan.

## Supplementary Materials

The following supporting information can be downloaded at: https://www.mdpi.com/article/10.3390/jcm12185835/s1, Table S1: Comparison of the two groups of patients with and without signs of pneumonia at admission.
