# Supplementary material for: Risk Factors for Post-Stroke Pneumonia in a Patient Population with Subacute Stroke: A Retrospective Cohort Study
Source: J Clin Med. 2023 Sep 8;12(18):5835. doi: 10.3390/jcm12185835 (PMC10532161; doi:10.3390/jcm12185835)
Supplement: Supplementary file 1 [file jcm-12-05835-s001.zip › jcm-2582138-supplementary.pdf]

Supplementary Material

In 340 subacute stroke patients, a comparison of two groups of patients, with and without signs of pneumonia at admission, was performed using the *t*-test, χ^2 test, and Mann-Whitney's U test, with the significance level set at 5%.

There were significant differences in age, sex (male), duration from stroke onset to admission, m-FIM at admission, FOIS at admission, and Alb levels between the group with and without pneumonia on chest CT at admission. In addition, the group with signs of pneumonia was significantly more likely to be transported to an emergency hospital during the hospitalization period due to worsening condition.

Table S1. Comparison of the two groups of patients with and without signs of pneumonia at admission.

	**Signs of pneumonia (n = 30)**	**No signs of pneumonia (n = 310)**	***p*-Value**
Age (y)	76.3(6.82)	70.0(14.6)	0.021
Sex (male) (%)	23(76.7)	178(52.4)	0.041
Duration from stroke onset to admission (days)	43.4(32.0)	33.2(19.8)	0.012
Length of stay (days)	89.3(56.2)	83.4(51.5)	0.552
Functional Independence Measure motor at admission	27.9(15.6)	42.3(21.4)	<0.001
Functional Independence Measure cognitive at admission	17.7(8.06)	20.7(8.79)	0.062
Stroke Impairment Assessment Set at admission	12.8(9.63)	15.7(8.76)	0.085
Functional Oral Intake Scale score at admission	4[1-5]	5[5-7]	<0.001
Body mass index at admission (kg/m^2)	20.8(2.25)	21.9(3.61)	0.112
White blood count at admission (°C)	7555.6(2109.2)	6781.2(2255.3)	0.072
C-reactive protein at admission (/μL)	1.11(1.51)	0.75(1.97)	0.243
Serum albumin at admission (mg/dl)	3.56(0.41)	3.85(0.46)	0.001
Transfer to acute care hospital due to worsening condition (%)	9(30.0)	44(12.9)	0.023

Quantitative variables are summarized as mean (standard deviation; SD) and median [interquartile range; IQR, 25–75th percentiles] values. Categorical variables are summarized as frequencies and percentages.
